# The effects of a sleep‐focused smartphone application on insomnia and depressive symptoms: a randomised controlled trial and mediation analysis

**DOI:** 10.1111/jcpp.13795

**Published:** 2023-03-29

**Authors:** Aliza Werner‐Seidler, Sophie H. Li, Samantha Spanos, Lara Johnston, Bridianne O'Dea, Michelle Torok, Lee Ritterband, Jill M. Newby, Andrew J. Mackinnon, Helen Christensen

**Affiliations:** ^1^ Black Dog Institute University of New South Wales Sydney NSW Australia; ^2^ School of Psychology University of New South Wales Sydney NSW Australia; ^3^ Australian Institute of Health Innovation Macquarie University Sydney NSW Australia; ^4^ School of Medicine University of Virginia Charlottesville VA USA

**Keywords:** Sleep disturbance, insomnia, adolescent, depression, app‐based intervention

## Abstract

**Background:**

Rates of depression are increasing among adolescents. A novel way to reduce depression is by improving sleep. We evaluated whether an app‐based intervention for insomnia improved sleep and depression, and whether changes in insomnia mediated changes in depression.

**Methods:**

We conducted a 2‐arm single‐blind randomised controlled trial at the Black Dog Institute in Australia. Adolescents 12–16 years experiencing insomnia symptoms were randomly allocated to receive Sleep Ninja, an app‐delivered cognitive behavioural therapy program for insomnia, or to an active control group involving weekly text message sleep tips. Assessments took place at baseline, 6 weeks (post‐intervention) and 14 weeks (post‐baseline). Co‐primary outcomes were symptoms of insomnia and depression at post‐intervention (primary endpoint). Intent‐to‐treat analyses were conducted. The trial is registered with the Australian and New Zealand Clinical Trials Registry, number ACTRN12619001462178.

**Results:**

Between October 25, 2019, and September 6, 2020, 264 participants were randomised to receive Sleep Ninja (*n* = 131) or to the control group (*n* = 133). Relative to the control group, those allocated to the intervention reported a greater reduction in insomnia symptoms at 6 weeks (95% CI: −2.96 to −0.41, *d* = .41) and 14 weeks (95% CI: −3.34 to −0.19, *d* = .39), and a greater reduction in depression symptoms at 6 weeks (95% CI: −3.46 to −0.56, *d* = .28) but not 14 weeks (*p* < 1). Change in insomnia mediated change in depression. No adverse events were reported.

**Conclusions:**

An app‐delivered program for insomnia could be a practical, non‐stigmatising and scalable way to reduce symptoms of insomnia and depression among adolescents experiencing difficulties getting enough good quality sleep.

## Introduction

Adolescence is a developmental period associated with a sharp rise in the onset of common mental disorders including depression (World Health Organisation, 2014). Moreover, rates of depression are increasing more rapidly in young people compared to adults, with approximately 13.2% of adolescents aged 12–17 years experiencing a major depressive episode in a one‐year period (Twenge, Cooper, Joiner, Duffy, & Binau, 2019). These factors suggest that intervention during this developmental period is critical. However, most young people with depression remain untreated (Islam, Khanam, & Kabir, 2020), due to a range of barriers including stigma, embarrassment, and a preference to manage the problem alone (Gulliver, Griffiths, & Christensen, 2010). An alternative pathway to addressing adolescent depression is via prevention and early intervention approaches. While up to 21% of depression cases could be prevented using currently available interventions (van Zoonen et al., 2014) the potential of these programs have been hampered by low uptake, limited accessibility and poor engagement (Lillevoll, Vangberg, Griffiths, Waterloo, & Eisemann, 2014). In many cases, adolescents without current symptoms are not motivated to engage in prevention programs potentially because there is no tangible or immediate benefit (Spence & Shortt, 2007). An approach that targets a self‐identified, existing and, ideally, non‐stigmatising problem (which is also a risk factor for depression), may be a better therapeutic candidate. This approach could prevent or treat depression, while simultaneously targeting a related problem. Sleep disturbance is one such factor: it is reported by 25%–66% of adolescents (Ohayon, Roberts, Zulley, Smirne, & Priest, 2000; Short, Gradisar, Gill, & Camfferman, 2013).

Depression and sleep are closely associated; with insomnia, the most common sleep disorder being a powerful risk factor for the onset of depression (Baglioni et al., 2011). Insomnia involves difficulty initiating or maintaining sleep with an associated impairment in daytime functioning (American Psychiatric Association, 2013). It affects approximately 4% of adolescents, with a further 25% of young people reporting subthreshold insomnia symptoms (Ohayon et al., 2000). Non‐depressed individuals with clinical levels of insomnia, are twice as likely to develop depression compared to those without insomnia (Baglioni et al., 2011). The association between sleep and depression is further underscored by studies of adults showing that insomnia‐focused treatments are associated with improvements in *both* insomnia and depression (Ashworth et al., 2015; Ritterband et al., 2009), and that reductions in depression are mediated by improvements in insomnia (Henry et al., 2021). Therefore, interventions that improve insomnia stand a good chance of reducing depression, which is supported by the adult literature (Gebara et al., 2018). One trial showed that improving sleep leads to reductions in depressive symptoms (Freeman et al., 2017), while another showed that treating insomnia in a previously depressed sample prevents the onset of new depressive symptoms (Christensen et al., 2016). Investigating whether these benefits extend to adolescents is needed, particularly given that sleep problems do not carry the same stigma that other psychiatric disorders do (Ribeiro et al., 2011) and therefore may be a more acceptable vector for intervention.

The most effective treatment for insomnia is Cognitive Behavioural Therapy for Insomnia (CBT‐I), which has superior outcomes in comparison to pharmacological treatments (Riemann et al., 2017) and is the gold standard treatment for insomnia. CBT‐I has customarily been provided face‐to‐face (Luik, Kyle, & Espie, 2017). However, digital delivery methods, such as web platforms and smartphone applications, have an accumulating evidence base supporting their use (Ritterband et al., 2009). Digital delivery overcomes obstacles including treatment accessibility, availability, variable clinician expertise, cost and stigma.

To date, two uncontrolled trials and one randomised controlled trial (RCT) have evaluated digital CBT‐I programs for adolescents with insomnia symptoms. Both uncontrolled trials demonstrated a reduction in insomnia symptoms, depression and anxiety following completion of the intervention (Cliffe, Croker, Denne, Smith, & Stallard, 2020; Werner‐Seidler et al., 2019). One of these trials used an app‐based intervention designed for adults in an adolescent sample (Cliffe et al., 2020), while the other was a pilot trial for the app‐based Sleep Ninja program (Werner‐Seidler et al., 2019). The RCT tested a therapist‐supported web intervention and found significant reductions in insomnia symptoms relative to a waitlist group (de Bruin, Oort, Bögels, & Meijer, 2014). These trials are promising but are limited in several respects. First, the RCT investigated a therapist‐supported intervention, and whether an automated intervention without support is effective in this age group is an unknown yet critical factor from the perspective of scaling the intervention. Second, these trials utilised small samples (ranging from *N* = 38–50/group). Third, the sole RCT involved a non‐active wait‐list control group. An RCT with an active control group will provide robust information about the effectiveness of an automated digital insomnia intervention for adolescents, over and above any placebo effects. Moreover, the mechanisms mediating change in insomnia and depression have not been investigated in adolescents. Knowledge about this relationship would advance our theoretical understanding of this link, as well as inform clinical practice.

In the first trial of its kind, the primary aim of the current study was to evaluate the effects of an automated app‐based insomnia intervention (Sleep Ninja) on adolescent insomnia and depressive symptoms relative to an active control group. Secondary aims were to investigate whether reductions in depression are mediated by improvements in insomnia, and to examine the effects of the intervention on anxiety symptoms, sleep quality, fatigue, daytime sleepiness, wellbeing, sleep‐related behaviours, dysfunctional beliefs about sleep and pre‐sleep arousal. Relative to an active control group, we hypothesised that the intervention group would be associated with better sleep, reductions in insomnia symptoms, reductions in depression and anxiety, and an improvement in sleep‐related behaviour and cognitions.

## Method

This trial was prospectively registered on the Australian New Zealand Clinical Trials Registry in October 2019 (#ACTRN12619001462178).

### Study design and participants

This was a single‐blind RCT with two parallel arms (intervention, active control) with 1:1 allocation, with a primary endpoint of six‐weeks (post‐intervention) and a 14‐week (post‐baseline) follow‐up assessment. Recruitment, screening, consent, assessments, allocation to condition and delivery of the intervention were carried out using an automated trial management system hosted by the Black Dog Institute. Participants were recruited into the study through online channels (paid, targeted social media advertisements on Facebook, Instagram and Twitter) and through community pathways (schools, psychology clinics, councils). Young people and their parents/guardians were invited to express interest in response to advertisements and were directed to the study website. The website provided information sheets and consent forms which were emailed back to the team. Active consent was required from both the young person and a parent/guardian to access the screening stage.

For inclusion, participants needed to be between the age of 12–16 years, report symptoms of at least subthreshold insomnia (score of ≥10 on the Insomnia Severity Index) (Bastien, Vallières, & Morin, 2001), own a smartphone (iPhone or Samsung), live in Australia, speak and read English fluently, provide parental and personal consent, and not have participated in pilot testing of the intervention (Werner‐Seidler et al., 2019). Participants were not excluded if they had severe sleep disturbances, suicidality or other mental health problems. To support trial safety and minimise risk, we used a risk management protocol whereby young people who reported suicidality were provided crises help lines they could contact and were offered a call back from the study psychologist. The purpose of these calls was to assist young people to access support services as appropriate, and not to provide therapeutic intervention. Participants could also engage in their usual treatment during the trial. Participants who met these criteria were invited into the trial. Recruitment commenced on the 25th of October 2019 and concluded on the 6th of September 2020. Final follow‐up data were collected on the 24 January, 2021.

### Randomisation and masking

Randomisation was performed by an automated computer‐generated program integrated into trial management software. Investigators were blinded to group allocation (except for the trial manager), and participants completed all assessments independently and online, without any contact with the research team.

### Measures

#### Primary outcomes

There were two co‐primary outcomes: insomnia symptoms and depression symptoms. The seven‐item Insomnia Severity Index (ISI) assesses insomnia symptoms over the previous 2 weeks (Bastien et al., 2001). Items are rated on a Likert scale ranging from 0 (none) to 4 (very severe), producing total scores of 0–28. Scores of 8–14 indicate subthreshold insomnia and 15–28 indicate clinical insomnia. The ISI was chosen as it is has been validated in adolescents with strong reliability (α = .83) and test–retest reliability (*r* = .79) (Chung, Kan, & Yeung, 2011).

The Patient Health Questionnaire‐Adolescent Version (PHQ‐A) (Johnson, Harris, Spitzer, & Williams, 2002) is a widely administered, nine‐item questionnaire assessing depressive symptoms in the preceding two weeks, adapted for adolescents. It has demonstrated strong psychometric properties, including internal consistency of α = .89, test–retest reliability of *r* = .84 (Kroenke, Spitzer & Williams, 2001), and high specificity (94%) and good sensitivity (73%) for major depressive disorder (Johnson et al., 2002). Items are scored on a 4‐point scale from 0 (not at all) to 3 (nearly every day). Total depression scores range from 0–27, with 10–14 indicating moderate symptoms, 15–19 indicating moderately severe symptoms and 20 and above indicating severe depression.

#### Secondary outcomes

Secondary outcome measures included anxiety, sleep quality, fatigue, sleepiness, wellbeing, sleep‐related behaviours, beliefs about sleep and pre‐sleep arousal. Details about these measures are reported in Appendix S1. Intervention adherence was measured as the number of lessons completed (out of six).

### Sample size

Sample size was calculated based on the smaller anticipated effect size of the co‐primary outcomes, depression, with an effect size of *d* = .30 based on a range of interventions including CBT in adolescent prevention depression studies (Werner‐Seidler et al., 2021). For 80% power, and α = .05 (2‐tailed), the required sample was 236, or 118 in each arm assuming a correlation of 0.50 between baseline and endpoint. Allowing an attrition rate of up to 30%, our target was 308 participants in total.

### Conditions – intervention and control

**Figure 1:**
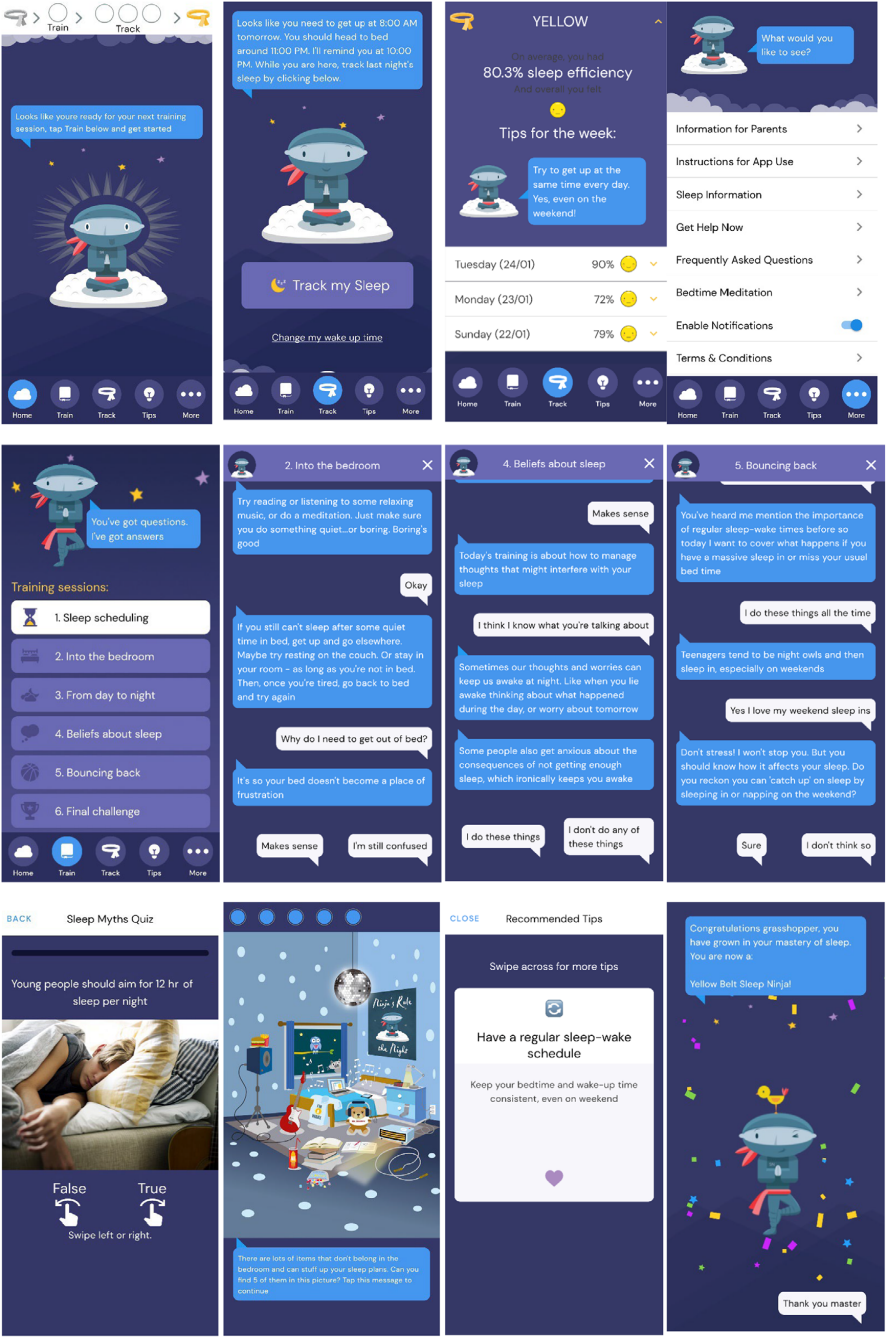
Top row from left shows Home screen, Sleep tracking screen, Sleep tracking results, More information section. Second row shows core therapeutic content. From left shows Training sessions, Example screen from ‘Into the bedroom’, Example screen from ‘Beliefs about sleep’, Example screen from ‘Bouncing back’. Third row shows games and engagement components. From left: Sleep myths quiz example (embedded in the ‘Sleep scheduling’ training session), Bedroom game where user has to identify and click on items in the room which might hinder sleep (embedded in ‘Into the bedroom’ training session), an example of a sleep tip, and a Level up screen example when moving to the next stage/belt colour level in the training sessions sequence [Color figure can be viewed at wileyonlinelibrary.com]

Intervention: The Sleep Ninja smartphone app is a CBT‐I intervention designed with the input of adolescents (Werner‐Seidler et al., 2017). Six training sessions (lessons) are delivered via the Sleep Ninja which acts as a sleep coach (see Figure 1). Each training session is 5–10 min in duration, covering: (a) psychoeducation, circadian rhythms, sleep–wake cycles; (b) stimulus control, guidelines for a wind‐down routine before bed; (c) sleep hygiene for evenings and daytime; (d) identification and planning for high‐risk situations and diversions from sleep routine; (e) cognitive therapy including how to deal with thoughts that can prevent sleep onset (e.g., repetitive worrying) and unhelpful beliefs about sleep; (f) a summary of the program and relapse prevention. Gamification principles are incorporated into the program as users progress to the next level (training session) and reach their next “belt” by completing one training session and tracking their sleep for 3 nights (out of a 7‐night period). This unlocks the next session and continues until they attain their “black belt” in sleep. A ‘Sleep Tips’ section of the app provides general sleep hygiene tips divided into tips for use during the day, before bed, or while trying to go to sleep. Users can scroll through the tips and ‘like’ those that they would like to save and revisit. There is a ‘Get Help Now’ section which lists crisis support lines. See Figure 1 for example app screens.

#### Control

The control group received weekly text messages for 6 weeks (matched to intervention period) that included tips and suggestions to improve sleep taken directly from the ‘Sleep Tips’ section of the Sleep Ninja app (see above). The control group material incorporates educational but non‐therapeutic components of the app, thereby offering an active control educational comparison and brief weekly engagement and interaction.

### Ethical considerations

All procedures were approved by the University of New South Wales Human Research Ethics Committee (HC#190139).

### Procedure

Participants completed consent forms and progressed through the screening process. Eligible participants proceeded to the baseline assessment and completed the questionnaire battery containing the ISI and PHQ‐9. The battery also contained additional outcomes (e.g., anxiety, wellbeing, fatigue), detailed in Appendix S1. Upon completion of the baseline measures, participants were randomised to the intervention or control condition using an automated computer‐generated program integrated into trial management software, with a 1:1 ratio. Participants allocated to the intervention group were directed via email and SMS to the App Store or Google Play to download the Sleep Ninja app onto their personal smartphone, using their unique trial registration details. They then had access to the app for 6 weeks. Participants allocated to the control condition were automatically informed via email and SMS that they would be receiving weekly sleep tips and suggestions to improve their sleep by SMS, for 6 weeks. They were also told that they would receive access to the Sleep Ninja intervention in a few months' time.

The post‐intervention assessment was administered following the 6‐week intervention period, and the follow‐up assessment was administered 2 months after the completion of the post‐intervention assessment. Participants in both groups were reimbursed with a $10 electronic gift card for completing questionnaires and sleep diaries at each assessment point (total of $30 for participating in the study).

### Statistical analyses

To examine intervention effects on insomnia and depression, ISI and PHQ‐A scores were examined using mixed‐model repeated measures (MMRM), with time as the within‐groups factor (baseline, post and follow‐up) and condition as the between‐group factor (intervention vs. control). This approach handles missing data by including all available data from each subject into the analysis and assumes missing data are missing at random. Given the purpose of the study, we were interested in the interaction effect between time and group (i.e., differences between the intervention and control groups in changes from baseline to post, and baseline to follow‐up). An unstructured covariance matrix accommodated within‐participant dependency, and degrees of freedom were estimated using Kenward‐Roger method. Between‐groups effect sizes were calculated as the modelled standardised mean difference at each occasion of measurement.

Analysis of the proportion of participants meeting the clinical threshold for caseness on the diagnostic measures (ISI, PHQ‐9) was assessed using mixed effects logistic regression model including participant as a random intercept effect. Caseness was determined by using accepted clinical cut‐offs for insomnia and depression (>14 on the ISI and >9 on the PHQ‐9).

Additionally, to analyse the effects of engagement, participants in the intervention group were categorised into high (completed ≥3 lessons) or low (completed 0–2 lessons) adherers and the analysis was repeated using adherence (high vs. low vs. control) as a between‐group factor.

To determine whether post treatment depressive symptoms were plausibly mediated by changes in insomnia, a model in which assignment to the intervention or control predicted post‐intervention depression (PHQ‐A) both directly and via post treatment insomnia severity (ISI) was fitted to participants with data on both occasions (baseline and post). Post‐intervention outcomes were adjusted for both baseline insomnia severity and depression. This model was using the Stata *sem* routine with bias corrected confidence intervals estimated from 10,000 bootstrap samples. All analyses were conducted using Stata 14 and statistical significance was set at α = .05.

## Results

### Participant characteristics

**Figure 2:**
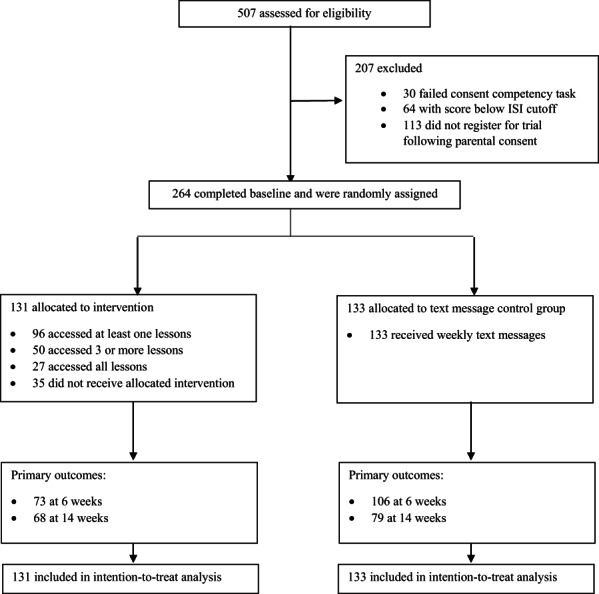
Trial profile. ISI, Insomnia Severity Index

Between October 25, 2019, and September 6, 2020, 264 participants were randomised to the Sleep Ninja intervention (*n* = 131) or to the control group (*n* = 133). The overall sample had a mean age of 14.71 years (*SD* = 1.21), with more females (71.3%) in the study relative to males. Participants were predominantly born in Australia (94%), which is higher than general population estimates which suggest that 84% of young people are born in Australia (Australian Bureau of Statistics, 2022). At baseline, participants showed elevated levels of depression (*M* = 13.95; *SD =* 5.83) and anxiety symptoms (*M* = 11; *SD =* 5.06) both of which are in the moderate range. General population estimates for this age group typically fall in the normal to mild range (Andreas & Brunborg, 2017; Tiirikainen, Haravuori, Ranta, Kaltiala‐Heino, & Marttunen, 2019), indicating the sample in this study were not representative of the general population, demonstrating higher comorbidity levels. Of participants recruited at baseline, 179 (68%) completed the primary outcome measures after the 6‐week intervention, and 147 (56%) completed the 2‐month follow‐up assessments (see Figure 2). Demographic characteristics by group are shown in Table 1.

**Table 1:** Participant characteristics

	Sleep Ninja intervention (*n* = 131)	Text message control (*n* = 133)
Age in years, mean (*SD*, range)	14.70 (1.16, 12.05–16.92)	14.74 (1.27, 12.02–16.97)
School grade, *n* (%)		
Grade 7	19 (14.4)	16 (12.0)
Grade 8	23 (17.4)	21 (15.8)
Grade 9	39 (29.5)	34 (25.6)
Grade 10	30 (22.7)	33 (24.8)
Grade 11	17 (12.9)	22 (16.5)
Other	4 (3.0)	7 (5.3)
Sex, *n* (%)		
Male	40 (30.3)	32 (24.1)
Female	89 (67.4)	100 (75.2)
Other	3 (2.3)	1 (0.8)
Born in Australia, *n* (%)	122 (92.4)	127 (95.5)
Previous diagnosis of depression or anxiety by a professional, *n* (%)	56 (42.4)	63 (47.4)
Currently receiving psychological treatment for sleep or mental health problem, *n* (%)	46 (34.8)	43 (32.3)
Taking medication, *n* (%)	38 (28.8)	46 (34.6)
Symptoms (*M*, *SD*)		
Insomnia; ISI	16.64 (4.11)	17.03 (3.58)
Depression; PHQ‐9	13.82 (5.84)	13.68 (5.85)
Anxiety; GAD‐7	10.98 (5.26)	11.01 (4.87)
Sleep quality: PQSI	10.87 (3.70)	11.23 (3.34)
Fatigue; FFS	17.85 (5.75)	16.84 (5.93)
Daytime sleepiness; ESS	8.01 (5.00)	8.23 (4.85)
Wellbeing; SWEMWBS	19.31 (4.80)	19.00 (4.20)
Sleep variables (*M*, *SD*)		
Sleep‐related behaviours; SRBQ	55.08 (18.12)	55.31 (18.13)
Dysfunctional beliefs and attitudes about sleep; DBAS	33.45 (5.99)	32.38 (5.99)
Pre‐sleep arousal; PSAS	48.64 (11.85)	49.83 (11.40)

### Primary and secondary outcomes

**Figure 3:**
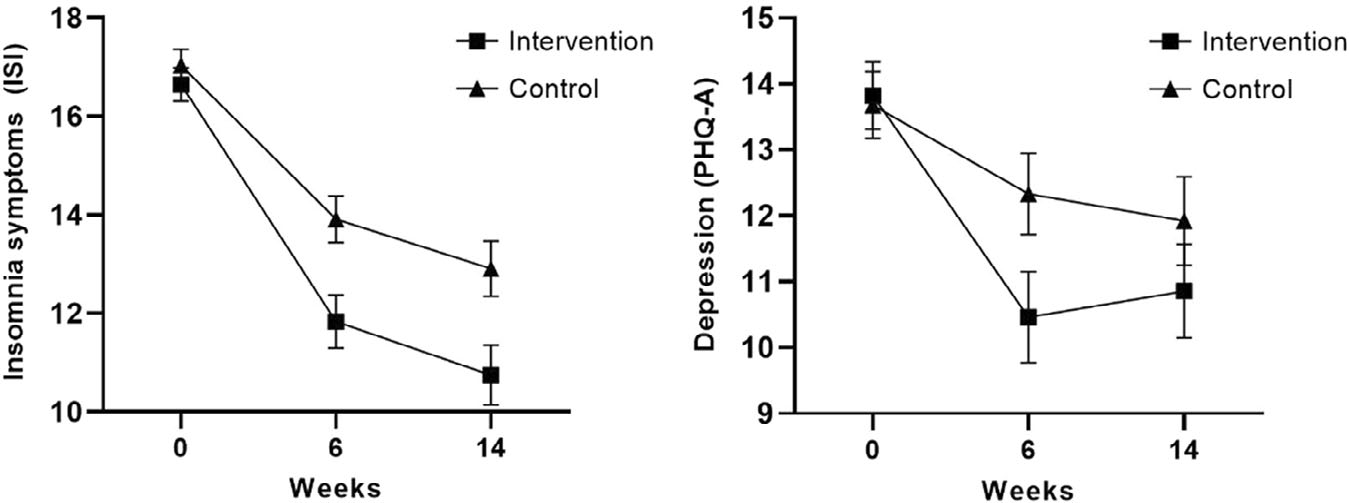
Left panel: Comparison of ISI insomnia estimated marginal means at baseline, 6 weeks (post‐intervention) and 14 weeks (follow‐up); Right panel: Comparison of PHQ‐A depression estimated marginal means at baseline, 6 weeks (post‐intervention) and 14 weeks (follow‐up). Error bars are ±1 standard error

Estimated marginal means and between‐group effect sizes are presented in Table 2. There was a significantly greater reduction in insomnia symptoms in the intervention group relative to the control group, post intervention, 95% CI: −2.96 to 0.41, *d* = .41, and follow up, 95% CI: −3.34 to −0.19, *d* = .39. Depression symptoms also showed a greater reduction from baseline in participants receiving the intervention compared to those in the control group at post, 95% CI: −3.46 to −0.56, *d* = .28, but not follow‐up (*p* > .05). See Figure 3. This analysis was repeated with the sleep item removed from the PHQ‐A, and outcomes were unchanged.

**Table 2:** Estimated marginal means and standard error for primary and secondary outcomes at each time point by group

	Sleep Ninja intervention			Text message control			Post‐assessment			2 Month follow‐up		
							Time × Group			Time × Group		
	Baseline (*n* = 132)	Post (*n* = 73)	2 months (*n* = 68)	Baseline (*n* = 133)	Post (*n* = 106)	2 months FU (*n* = 79)	*t*	*df*	*p*	*t*	*df*	*p*
Primary outcomes												
Insomnia; ISI	16.64 (0.34)	11.83 (0.54)	10.74 (0.61)	17.03 (0.33)	13.91 (0.47)	12.90 (0.56)	−2.60	195.0	.01*	−2.21	172.8	.03*
Depression; PHQ‐A	13.82 (0.51)	10.46 (0.69)	10.86 (0.71)	13.68 (0.51)	12.33 (0.62)	11.92 (0.67)	−2.73	185.6	.01*	−1.36	164.1	.17
Secondary outcomes												
Anxiety; GAD‐7	10.98 (0.44)	8.76 (0.61)	9.18 (0.63)	11.01 (0.44)	10.33 (0.55)	10.01 (0.59)	−2.29	183.5	.02*	−1.06	162.8	.29
Sleep quality; PQSI	10.87 (0.31)	9.01 (0.46)	8.42 (0.48)	11.26 (0.31)	10.56 (0.42)	8.42 (0.48)	−2.03	135.3	.04*	−2.26	132.4	.03*
Fatigue; FFS	17.85 (0.51)	15.12 (0.73)	14.71 (0.87)	16.81 (0.51)	15.43 (0.63)	14.16 (0.77)	−1.60	169.2	.11	−0.44	138.9	.66
Daytime sleepiness; ESS	8.01 (0.42)	7.42 (0.53)	6.82 (0.59)	8.23 (0.43)	7.15 (0.46)	6.80 (0.52)	0.82	173.9	.42	0.33	138.0	.74
Wellbeing; SWEMWBS	19.31 (0.39)	21.13 (0.52)	21.21 (0.58)	19.00 (0.39)	20.16 (0.46)	20.17 (0.52)	1.06	185.5	.29	1.01	155.3	.31
Sleep‐related behaviours; SRBQ	55.08 (1.58)	51.50 (1.95)	45.95 (2.38)	55.32 (1.57)	52.15 (1.69)	50.28 (2.10)	−0.18	178.5	.86	−1.35	141.8	.18
Dysfunctional beliefs and attitudes about sleep; DBAS	33.45 (0.52)	30.50 (0.70)	27.64 (0.90)	32.38 (0.52)	31.28 (0.59)	30.47 (0.79)	−2.08	180.9	.04*	−3.35	133.4	.01*
Pre‐sleep arousal; PSAS	48.63 (1.01)	42.19 (1.35)	42.53(1.55)	49.83 (1.01)	48.62 (1.20)	46.93 (1.38)	−3.67	167.5	.01*	−1.72	135.5	.09

*All *p* values rounded to two decimal places.

The same pattern was found for anxiety symptoms, with a greater reduction from baseline to post in the intervention group relative to the control group, 95% CI: −2.89 to −0.21, *d* = .27, but not at follow‐up (*p* > .05). Results from the analysis of sleep quality demonstrated a greater improvement from baseline to post, 95% CI: −2.30 to −0.03, *d* = .39 and at follow‐up, 95% CI: −2.45 to −0.16, *d* = .44, in the intervention group relative to the control group. There was also an effect of dysfunctional beliefs about sleep, with a greater reduction in these beliefs among the intervention group at post, 95% CI: −3.36 to −0.09, *d* = .13, and follow‐up, 95% CI: −6.21 to −1.60, *d* = .40, relative to the control group. Finally, there was an effect at post‐intervention for pre‐sleep arousal, with a greater reduction of arousal in the intervention group relative to control, 95% CI: −8.05 to −2.42, *d* = .10, but not at follow‐up. There were no effects for fatigue, daytime sleepiness, wellbeing or sleep‐related behaviours (all *p*s > .05).

### Clinical status post assessment

As shown in Table 3, based on mixed effects logistic models, there were significant group differences in the proportion of individuals who met the clinical cut‐off thresholds from baseline to post‐intervention for depression (*z* = 3.41, *p* < .001). While between group change was not significant for sleep outcomes, (*z* = 1.87, *p* = .06), predicted caseness was significantly lower at post‐intervention for Sleep Ninja (*z* = 2.99, *p* = .00). At follow‐up, differences in probable depression caseness remained significant (*z* = 1.97, *p* = .049).

**Table 3:** Estimated prevalence (and 95% confidence intervals) of participants meeting clinical cut‐off thresholds at baseline, post intervention and at follow‐up

	Intervention			Active control			*p* Values^a^	
	Baseline (*n* = 131)	Post (*n* = 74)	FU (*n* = 70)	Baseline (*n* = 133)	Post (*n* = 106)	FU (*n* = 79)	Post	FU
Insomnia (15+ on ISI)	70.2% (62.4%–78.0%)	26.7% (17.1%–36.3%)	30.9% (20.6%–41.3%)	74.8% (67.4%–82.1%)	47.0% (37.8%–56.2%)	34.3% (24.3%–44.4%)	.061	.851
Depression (10+ on PHQ‐A)	76.7% (69.6%–83.8%)	44.3% (33.5%–55.0%)	52.7% (41.8%–63.7%)	70.2% (62.4%–78.1%)	63.7% (54.7%–72.7%)	61.0% (50.9%–71.0%)	.001	.049

^a^ Tests of differential change from baseline in logistic model with random participant intercept.

### Attrition, adherence and intervention effects

Post intervention, 70% of participants completed the primary outcome questionnaires (56% in the intervention group and 83% in the control group). At follow‐up, 56% of participants completed these questionnaires (52% in the intervention group and 59% in the control group). See Figure 2. An analysis of demographic characteristics and baseline symptoms as predictors of attrition was conducted. Univariable logistic regressions were conducted for all participants, with an overall effect of ISI (*p* = .03), and an interaction effect between group and age (*p* = .01), and group and school grade (*p* = .03), both in the intervention group. A subsequent model which included the significant variables and terms (ISI and ISI × age) indicated that in the intervention group, older age was a significant predictor of attrition, while insomnia scores at baseline had a negligible effect.

In terms of adherence to the intervention, participants in the intervention condition completed an average of 2.30 (38%) of the six Sleep Ninja modules, with 35 (27%) not completing any lessons, 46 (35%) completing between one‐to‐two lessons, 50 (38%) completing three or more lessons and of these, 27 (21%) completing all six lessons. An analysis of demographic characteristics and baseline symptoms as predictors of adherence was conducted in the intervention arm. Participants were dichotomised into high and low adherers, and a logistic regression was conducted. Results indicated that age was the only significant predictor of adherence, with older age predicting completion of fewer modules (*p* = .03).

A significant interaction effect between adherence and intervention effects indicated that adherence to the Sleep Ninja intervention affected outcomes. Reductions in insomnia symptoms from baseline was greater for high adherers compared to control group participants both post intervention [*t*(189.1) = −2.62, *p* = .010, *d* = .50] and at follow‐up [*t*(168.4) = −3.22, *p* = .001, *d* = .67]. Effect sizes were 0.50 (95% CI 0.15 to 0.85) post intervention and 0.67 (95% CI 0.29 to 1.04) at follow‐up. No significant differences in insomnia symptom reduction were evident between the low adherers and control group on either measurement occasion (*p*s > .05); effect sizes were 0.32 (95% CI −0.03 to 0.68) at post intervention and 0.10 (95% CI −0.28 to 0.47) at follow‐up. The same pattern of results was evident for depression symptoms, with high adherers showing greater reductions from baseline at post [*t*(181.5) = −2.95, *p* = .00, *d* = .46, 95% CI 0.11 to 0.80] and follow‐up [*t*(160.6) = −2.48, *p* = .01, *d* = .48, 95% CI 0.11 to 0.85] compared to the control group, and no differences between low adheres and control participants at either measurement occasion (*p*s > .05).

### Mediation analysis

A mediation analysis was conducted to examine whether changes in insomnia mediated changes in depression. The total post‐intervention difference between Sleep Ninja and control was 0.29 standard deviations (*p* = .01, 95% CI: 0.07 to 0.51) on the PHQ‐A. Of this, 0.17 standard deviations or 59% of the total effect was attributable to a mediating effect of insomnia severity (*p* = .00, 95% CI: 0.07 to 0.30). The direct effect of the intervention on depression was 0.12 standard deviations or 41% of the total. This was not statistically significant (*p* = .22, 95% CI: −0.70 to 0.31). This pattern suggests that the effects of the intervention on depression may be occurring via insomnia severity. The direct effect of the intervention on insomnia remained significant in the presence of the indirect path through depression (effect: 0.21 standard deviations, *p* = .04, 95% CI: 0.01 to 0.41).

## Discussion

We found a greater reduction in insomnia symptoms in the Sleep Ninja group relative to the control, with a medium effect size. Adolescents allocated to the intervention also showed small reductions in symptoms of depression immediately after the intervention. This was confirmed in terms of caseness, with significantly fewer participants in the intervention group meeting criteria for probable depressive disorder following the intervention, relative to the control group (44% for the intervention group compared to 64% for the control group, respectively). Mediation analysis supported the hypothesis that the mechanism by which symptoms of depression are reduced is, at least in part, attributable to improvements in sleep (although causality cannot be attributed rigorously given the outcomes were assessed contemporaneously). This is the first trial, to our knowledge, that has investigated the effectiveness of an app‐delivered intervention targeting sleep in adolescents, on both insomnia and depression. These findings contribute to emerging evidence of positive effects of digital sleep interventions and indicate a mechanistic and potentially causal role for insomnia symptoms in depression (Christensen et al., 2016; Freeman et al., 2017). The current study extends the literature by establishing this effect in adolescents.

The effect sizes from this trial were in the small to medium range (*d* = .27–.41), which is within expectations given the non‐clinical environment and nature of the sample, and comparable to previous non‐clinical adolescent trials (Werner‐Seidler et al., 2021). At baseline, participants were at the lower end of the moderate level of insomnia and depression. This restricted symptom range (as compared to clinical groups) mean that large effects are unlikely to be detected. Interventions that deliver small to medium effect sizes can have substantial impact at a population level when delivered to large numbers of adolescents (Matthay et al., 2021).

This trial outcome is significant particularly given the brevity and simplicity of the intervention. As is common to most CBT‐I programs, core components of Sleep Ninja include psychoeducation, stimulus control, sleep hygiene, sleep‐focused cognitive therapy, program review and relapse prevention. Consultation with young people and their parents led to the deliberate omission of sleep restriction given the unguided format of the program, coupled with the age of participants (Werner‐Seidler et al., 2017). Given the strong evidence‐base underlying the effectiveness of sleep restriction (Spielman, Saskin, & Thorpy, 1987), it is encouraging that significant benefits were nonetheless found for Sleep Ninja, even without this component. Moreover, each module takes 5–10 min to complete, and the whole program, although sequentially spaced over a minimum of 3 weeks, is intended to be completed within a total of 1 hr. This is much briefer than traditional CBT‐I packages delivered face‐to‐face which generally require delivery over at least 6 hr (de Bruin et al., 2014) and briefer than digital adult CBT‐I programs (e.g., SHUTi, Sleepio), which require between 3–6 hr to complete (Espie et al., 2012; Ritterband et al., 2009). In the development stage, we balanced young people's preferences for a short intervention that could be consumed in small bite‐size components with the need to include sufficient therapeutic content for behavioural change (Werner‐Seidler et al., 2017). The results from the current trial suggest that such a balance was largely achieved. Although speculative, these results may indicate that when intervening at this age, adolescents are able to effectively absorb the key components of the intervention likely to lead to change.

Despite the brevity of the intervention, adherence and completion rates were modest. Over a quarter (27%) of participants allocated to the intervention group did not start the program at all, and 35% accessed only 1 or 2 lessons. Those who completed fewer than 3 lessons had comparable outcomes (sleep, depression, anxiety) to the control group, although caution is needed in the interpretation of this finding because dichotomising participants into high and low adherers violates randomisation. That said, adherence rates accord with studies investigating smartphone app use for mental health issues (Linardon & Fuller‐Tyszkiewicz, 2020), and the pattern of results linking therapeutic outcomes with engagement is consistent with the literature (Gan, McGillivray, Han, Christensen, & Torok, 2021). It is encouraging that overall, findings for the intervention group were positive despite a significant proportion of those in the intervention group not using the app extensively. At the same time, future research should focus on increasing intervention adherence to optimise outcomes.

The finding that younger participants completed more of the intervention suggests that this program might be more appealing and better suited to the younger age group (e.g., 12–14 year olds), and underscores the need to very specifically and purposefully tailor the intervention for particular ages. Indeed, what is likely to appeal to a 12‐year‐old is likely to differ to that of a 16‐year‐old. Similarly, this program may not be suitable or appealing to all adolescents experiencing insomnia. For example, there are some comorbidities in which CBT‐I strategies may not be appropriate, such as diabetes type 1 (Gregory et al., 2022) and some cases of Bipolar Disorder (Kaplan & Harvey, 2013). It is also recommended that this program be used in collaboration with health professionals for young people who experience complex sleep, mental health and/or chronic health conditions. Future research could examine whether the app could be used by clinicians treating young people for insomnia, depression and a range of comorbidities in clinical practice.

While the development of Sleep Ninja followed best‐practice guidelines for intervention development (e.g., involve end users at conception and design), finding ways to improve engagement is needed. One way to do this would be to explore whether adding in a human‐supported component improves adherence, as is case in other digital interventions (Clarke, Kuosmanen, & Barry, 2014). Identifying participant groups most likely to require and benefit from this human support would enable the delivery of targeted support given that not all participants will require this. For this app to be appropriate for use by diverse groups of young people (e.g., in terms of age, gender, culture, neurodiversity, comorbidities), piloting the program with young people from these groups, and adapting the app in collaboration with these young people and health professionals is necessary to confirm that the program is suitable for use and engaging.

There are several limitations associated with the current study. First, it involved a community sample recruited on the basis of experiencing insomnia symptoms. The effects observed on mental health represent a mix of prevention (sleep effects not reaching caseness), early intervention (early onset of symptoms) and treatment (case level) depending on participants' baseline illness status. Establishing Sleep Ninja as either a depression‐prevention program, or as a treatment program will require further trials. While the current design precludes formal conclusions regarding specific preventive or treatment effects of the program, the inclusion of a community sample likely makes the broad findings generalisable to young people across the symptom continuum. Second, while the active control group offers a more robust comparison relative to non‐active control groups (e.g., waitlist, no intervention), it was not completely matched to Sleep Ninja for time or attention, and so the contribution of these factors to the outcome cannot be ruled out. Finally, older participant age was associated with attrition, suggesting that the current intervention was less appealing to older adolescents. Whether older adolescents engage better with adult sleep interventions or need one especially designed for their age group will need to be addressed in the future.

This trial has shown, for the first time, the insomnia and mental health benefits associated with a brief, self‐guided intervention for use by young people. This study represents a shift in thinking about how depression might be addressed and suggests there is value in focusing on sleep‐focused treatment to improve adolescent mental health.

## Supporting information


**Appendix S1** Secondary outcomes.
