# Supplementary material for: The effects of a sleep‐focused smartphone application on insomnia and depressive symptoms: a randomised controlled trial and mediation analysis
Source: J Child Psychol Psychiatry. 2023 Mar 29;64(9):1324–35. doi: 10.1111/jcpp.13795 (PMC10952387; doi:10.1111/jcpp.13795)
Supplement: Supplementary file 1 — Appendix S1 Secondary outcomes. [file JCPP-64-1324-s001.docx]

Supporting information

*Appendix S1. Secondary Outcomes*

Anxiety was assessed using the Generalised Anxiety Disorder 7 (GAD-7) assesses symptoms of generalised anxiety disorder in the preceding two weeks (Spitzer, Kroenke, Williams & Lowe, 2006). The seven items are scored on a 4-point scale from 0 (not at all) to 3 (nearly every day). Total anxiety scores range from 0-21, with 10-14 indicating moderate anxiety and 15 and above indicating severe anxiety. The GAD-7 demonstrates strong internal consistency (α = 0·90), convergent and discriminant validity in adolescent populations (Tiirikainen, Haravuori, Ranta, Kaltiala-Heino & Marttunen, 2019).

Sleep quality was assessed using the Pittsburgh Sleep Quality Index (PSQI), which is a 19-item measure with scores ranging from 0-21, and strong psychometric properties in adolescents (Buysse, Reynolds, Monk, Berman & Kupfer, 1989; de la Vega et al., 2015).

Fatigue was assessed using the 7-item Flinders Fatigue Scale (FFS) which assesses fatigue over the previous two weeks, to produce a score between 0-31 (Gradisar et al., 2007).

Sleepiness was measured using the Epworth Sleepiness Scale for Children and Adolescents (ESS), which is an 8-item measure that assesses daytime sleepiness over the preceding month and produces a score between 0-24 (Janssen, Phillipson, O’Connor & Johns, 2017).

Wellbeing was measured using the Short Warwick-Edinburgh Mental Wellbeing Scale (SWEMWBS), a 7-item measure of wellbeing, strong psychometric properties and scores ranging from 7-35 (Tennant et al., 2007).

Sleep- behaviours were measured using the Sleep-Related Behaviours Questionnaire (SRBQ) which assesses unhelpful sleep-related thoughts and behaviour (Ree & Harvey, 2004). There are 32 items, and scores range from 0-128.

Attitudes and beliefs about sleep were measured using the Dysfunctional Beliefs and Attitudes about Sleep Scale for Children (DBAS). This 10-item measure assesses dysfunctional beliefs and attitudes about sleep with scores from 1-50 and strong psychometric properties (Blunden, Gregory & Crawford, 2013).

Arousal before bed was measured using the Pre-Sleep Arousal Scale (PSAS). This 16-item scale assesses somatic and cognitive arousal before bedtime, with scores between 16-80 (Nicassio, Mendlowitz, Fussell & Petras, 1985). A higher score on these measures reflects a higher level of the variable being measured.

References

Blunden, S., Gregory, A.M., & Crawford, M.R. (2013). Development of a short version of the Dysfunctional Beliefs about Sleep Questionnaire for use with Children (DBAS-C10). *Jouranl of Sleep Disorders, 6,* 8-10.

Buysse, D.J., Reynolds, C.F., Monk, T.H., Berman, S.R., & Kupfer, D.J. (1989). The Pittsburgh Sleep Quality Index: Anew instrument for psychiatric practice and research. *Psychiatry Research, 28,* 193–213.

de la Vega, R., Tome-Pires, C., Sole, E., Racine, M., Castarlenas, E., Jensen, M.P., & Miro, J. (2015). The Pittsburgh Sleep Quality Index: Validity and factor structure in young people. *Psychological Assessment, 27,* 22–7.

Gradisar, M., Lack, L., Richards, H., Harris, J., Gallasch, J., Boundy, M. & Johnston. (2007). The Flinders Fatigue Scale: Preliminary psychometric properties and clinical sensitivity of a new scale for measuring daytime fatigue associated with insomnia. *Journal of Clinical Sleep Medicine, 3,* 722–8.

Janssen, K.C., Phillipson, S., O'Connor, J., & Johns, M.W. (2017). Validation of the Epworth Sleepiness Scale for children and adolescents using rasch analysis. *Sleep Medicine, 33,* 30–5.

Nicassio, P.M., Mendlowitz, D.R., Fussell, J.J., & Petras, L. (1985). The phenomenology of the pre-sleep state: The development of the pre-sleep arousal scale. *Behaviour Research and Therapy, 23,* 263–71.

Ree, M.J., & Harvey, A.G. (2004). Investigating safety behaviours in insomnia: the development of the Sleep-related Behaviours Questionnaire (SRBQ). *Behaviour Change, 21,* 26–36.

Spitzer, R.L., Kroenke, K., Williams, J.B. & Lowe, B. (2006). A brief measure for assessing

generalized anxiety disorder: The GAD-7. *Archives of Internal Medicine*, *166,* 1092–7.

Tennant, R., Hiller, L., Fishwick, R., Platt, S., Joseph, S., Weich, S., Parkinson, J., Secker, J., & Stewart-Brown, S. (2007). The Warwick-Edinburgh Mental Well-being Scale (WEMWBS): development and UK validation. *Health and Quality of Life Outcomes, 5,* 63.

Tiirikainen, K., Haravuori, H., Ranta, K., Kaltiala-Heino, R., & Marttunen, M. (2019). Psychometricproperties of the 7-item Generalized Anxiety Disorder Scale (GAD-7) in a large representative sample of Finnish adolescents. *Psychiatry Research*, *272,* 30–5.
